# First Impression Misleads Emotion Recognition

**DOI:** 10.3389/fpsyg.2019.00527

**Published:** 2019-03-20

**Authors:** Valentina Colonnello, Paolo Maria Russo, Katia Mattarozzi

**Affiliations:** Department of Experimental, Diagnostic and Specialty Medicine, University of Bologna, Bologna, Italy

**Keywords:** emotion recognition, trustworthiness, social perception, interpersonal interaction, social behavior

## Abstract

Recognition of others’ emotions is a key life ability that guides one’s own choices and behavior, and it hinges on the recognition of others’ facial cues. Independent studies indicate that facial appearance-based evaluations affect social behavior, but little is known about how facial appearance-based trustworthiness evaluations influence the recognition of specific emotions. We tested the hypothesis that first impressions based on facial appearance affect the recognition of basic emotions. A total of 150 participants completed a dynamic emotion recognition task. In a within-subjects design, the participants viewed videos of individuals with trustworthy-looking, neutral, or untrustworthy-looking faces gradually and continuously displaying basic emotions (happiness, anger, fear, and sadness). The participants’ accuracy and speed in recognizing the emotions were measured. Untrustworthy-looking faces decreased participants’ emotion recognition accuracy and speed, across emotion types. In addition, faces that elicited a positive inference of trustworthiness enhanced emotion recognition speed of fear and sadness, emotional expressions that signal another’s distress and modulate prosocial behavior. These findings suggest that facial appearance-based inferences may interfere with the ability to accurately and rapidly recognize others’ basic emotions.

## Introduction

The accurate and fast recognition of others’ emotions is a key life ability that guides one’s own choices and actions (Elfenbein and Ambady, 2002). Such ability is a central component of fluent social interactions, and difficulties in accurately detecting others’ emotional perspectives are associated with poor interpersonal functioning (Shimokawa et al., 2001). The recognition of others’ emotions hinges on perception of facial expressions (Zebrowitz, 2006). Perceivers not only quickly make inferences about others’ current emotional experience, but they can also automatically and unintentionally make inferences about others’ personality traits by evaluating emotionally neutral faces (Todorov et al., 2015, for a review). There are circumstances in which the actual social interaction may follow a glimpse of a neutral picture of the other’s face (Todorov et al., 2005; Sussman et al., 2013; Tingley, 2014; Fruhen et al., 2015; Wilson and Rule, 2015).

Previous studies indicate that facial emotion recognition and the formation of trait impressions from facial appearance are two central and interconnected components of social interaction that rely on the same functional mechanisms, and both robustly affect behaviors and decisions by regulating adaptive appetitive/defensive responses (see Todorov et al., 2015, for a review). The relationship between recognition of subtle facial emotional expressions and first-impression inferential processes has been demonstrated (Todorov et al., 2015, for a review). Emotionally neutral faces rated at the extreme positive end of the trustworthiness dimension, which is the best approximation of valence evaluation (Oosterhof and Todorov, 2008; Todorov et al., 2015), are perceived as resembling facial expressions of happiness, and faces rated at the extreme negative end of that dimension are perceived as resembling facial expressions of anger (Oosterhof and Todorov, 2008, 2009). However, studies investigating whether faces differing for their trustworthiness appearance affect the accurate and fast recognition of specific positive and negative facial emotional expressions are scarce. The present study therefore aimed to scrutinize the effects of trustworthiness inferences on recognition of discrete emotional expressions, using a dynamic emotion recognition task.

We hypothesized that (1) changes in trustworthiness inferences from facial appearance modulate the ability to rapidly and accurately recognize facial expressions of emotions and (2) this effect differs for different emotional expressions.

Given that positive and negative trustworthiness judgments from facial appearance activate approach/avoidance behaviors respectively (Adolphs et al., 1998; Oosterhof and Todorov, 2008; Mattarozzi et al., 2017), we predicted a general recognition advantage for emotions expressed by trustworthy-looking faces and an emotion recognition disadvantage for the emotions expressed by untrustworthy-looking faces.

With respect to the recognition of specific emotions, given the perception of similarity between untrustworthy and angry faces and trustworthy and happy faces (Oosterhof and Todorov, 2008), it is plausible to expect that the inferences of trustworthiness from facial appearance can act as cue producing an affective priming and, in turn, enhance the recognition of happiness or anger when expressed, respectively, by trustworthy or untrustworthy-looking face.

Alternatively, in the light of behavioral (Oosterhof and Todorov, 2008) and functional neuroimaging (Winston et al., 2002; Engell et al., 2007) studies suggesting a threat effect of faces perceived as untrustworthy, it is possible that faces perceived as untrustworthy would capture more task-irrelevant attentive resources and, in turn, reduce the recognition of all emotional expressions.

In addition, given that fear and sadness may elicit approaching caring behavior (Marsh et al., 2005, 2007; Seidel et al., 2010, but see Adams and Kleck, 2003) and that faces perceived as trustworthy elicit caring motivation (Mattarozzi et al., 2017), it is possible to expect that positive inferences of trustworthiness may provide the perceptual and affective context that likely facilitates the recognition of such emotions.

## Materials and Methods

### Participants

A convenient sample of 150 nurse practitioners (27 men, 123 women; all Caucasians, age: *M* = 32.65, *SD* = 12.47 years; all having at least 12 months of experience as a nurse) were recruited from the Bologna University Hospital, Italy. The number of participants was more than the minimum required sample size to account for potential drop-outs. An *a priori* sample size calculation using G*Power software (Faul et al., 2007) indicated that a minimum of 43 participants were needed to achieve a statistical power of 0.95 for alpha = 0.05, assuming a medium effect size and a correlation of 0.50 between repeated measures.

The inclusion criteria were having normal or corrected vision and no self-reported physical conditions interfering with ability to complete a computer task. In a single wave of data collection, all potential participants were contacted by email and were asked to voluntarily participate in the present study. No participant dropped out or was excluded from the study.

All participants signed informed consent prior to the study and all were fully debriefed at the study’s conclusion. The experimental procedures were approved by the institutional review board (IRB) of the University of Bologna, Italy.

### Emotion Recognition Task

A total of 72 video clips (10 s each, 25 frames/s) were used as stimuli. Each video clip showed a neutral facial expression gradually and continuously changing into a basic full-intensity facial emotional expression (happiness, anger, fear, or sadness).

For construction of the video stimuli, 90 frontal, full-color images of the faces of 18 Caucasian actors were selected from the Karolinska Directed Faces Database (Lundqvist and Litton, 1998; http://www.emotionlab.se/kdef/). The images were selected based on a standardized average (*z* score) of their trustworthiness ratings, as in Oosterhof and Todorov (2008). Specifically, from the database available at http://tlab.princeton.edu/databases/karolinskafaces/, we selected the three male and three female faces rated as the most trustworthy-looking (*z* = +0.74 ± 0.22; faces: AM43; AM58; AM66; AF06; AF19; AF01), the three male and three female faces rated as neutral or moderately trustworthy (*z* = −0.02 ± 0.11; faces: AM45; AM64; AM70; AF20; AF28; AF32), and the three male and three female faces rated as the most untrustworthy-looking (*z* = −0.68 ± 0.042; faces: AF12; AF21; AF33; AM42; AM67; AM68).The images used for the practice trials had neutral trustworthiness *z* scores (*z* = −0.04 ± 0.2, faces: AM44; AF04). For each actor, we selected images representing a neutral emotional expression and four full-intensity emotional expressions (happiness, anger, fear, sadness). Two additional images presenting the neutral and full emotional expressions of two actors (one female and one male) were used to construct the videos for the practice trials.

**Figure 1:**
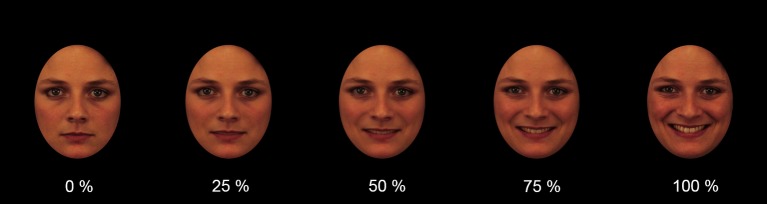
Example of video frames depicting a trustworthy-looking face gradually displaying the basic emotion of happiness.

Each image was manipulated to cover nonfacial attributes (e.g., ears, hair, and background). The software AbrosoftFantaMorph (http://www.fantamorph.com/index.html) was used to morph each image with neutral facial expression into an image with a full emotional expression, resulting in faces showing a change of emotional intensity from 0% (neutral) to 100% (emotion). Figure 1 depicts an example of a trustworthy-looking face gradually displaying happiness.

For each actor, four videos (neutral-happy, neutral-angry, neutral-fearful, and neutral-sad) were composed. The task consisted of 4 practice trials and 72 test trials. Each trial presentation was preceded by a central fixation cross (400–600 ms). The video clips presentation order was pseudorandomized controlling for facial appearance and emotion type presented: no more than two videos of faces with same perceived valence (trustworthy, neutral, and untrustworthy) and of the same emotion expression type (happiness, anger, fear, and sadness) were presented consecutively. All stimuli were centrally presented on a black background.

Participants were instructed to view each video and press the keyboard spacebar as soon as they felt certain that the image contained more of the features of a specific emotion than of the initial neutral facial expression. Immediately after stopping the video, the stopped frame remained visible on the center screen and the participant identified the displayed emotion by completing a forced-choice task recognition between four possible emotion labels (happiness, anger, fear, and sadness). There was no time limit or feedback throughout the task. The total duration of the task was ~20 min.

After presentation of the last video, a manipulation check was performed to determine whether participants’ judgments of trustworthiness were coherent with judgments of trustworthiness reported in the database. The 18 neutral facial expressions used for the initial frame of the videos (http://tlab.princeton.edu/databases/karolinskafaces/) were individually presented on the center screen, and participants were instructed to rate each face for perceived trustworthiness using a 9-point Likert scale (1 = “not at all” and 9 = “extremely”). For stimulus presentation and response data collection, we used E-Prime software (http://www.pstnet.com/).

### Statistical Analysis

The accuracy (the percentage of correct responses in the forced-choice emotion recognition) and the speed (time required to correctly recognize the emotions) data were analyzed using separate repeated-measures ANOVAs, with Facial Appearance (trustworthy, neutral, untrustworthy) and Emotion (happiness, anger, fear, sadness) as within-subject factors. The trustworthiness rating was analyzed using the ANOVA, with Facial Appearance (trustworthy, neutral, and untrustworthy) as a within-subject factor. ANOVAs were followed by Bonferroni’s *post hoc* analyses.

## Results

### Manipulation Check

The selected images were perceived as differing in appearance-based trustworthiness, *F*(2,98) = 49.48, *p* < 0.0001; $\eta_{p}^{2}=0.50.$ Consistent with trustworthiness judgments reported in the database, Bonferroni’s test indicated that trustworthy faces (*M* = 3.80, *SD* = 1.87) were rated more positively than neutral (*M* = 3.25, *SD* = 1.56) and untrustworthy faces (*M* = 2.41, *SD* = 1.43, all *p_s_* < 0.05).

### Accuracy

Participants’ overall accuracy in recognizing emotions was high (80.19% of correct responses). With respect to Emotions, *F*(3,447) = 100.41, *p* < 0.0001, $\eta_{p}^{2}=0.40,$ Bonferroni’s test revealed that happiness (*M* = 94.42%, *SD* = 12.13%) and fear (*M* = 74.65%, *SD* = 21.66%) were recognized the most and least accurately, respectively (*p_s_* < 0.05). Bonferroni’s test revealed no differences in recognition accuracy between angry (*M* = 79.45%, *SD* = 20.82%) and sad (*M* = 79.62%, *SD* = 20.47%) expressions (*p* > 0.05).

As revealed by Bonferroni’s test on the main effect of Facial Appearance, *F*(2,298) = 109.66, *p* < 0.0001, $\eta_{p}^{2}=0.42,$ emotions displayed by untrustworthy faces (*M* = 74.94%, *SD* = 21.94%) were recognized less accurately than emotions displayed by neutral (*M* = 86.07%, *SD* = 19.41%) and trustworthy (*M* = 85.84%, *SD* = 18.62%) faces (*p_s_* < 0.05), with no difference between the latter two groups (*p_s_* > 0.05).

**Figure 2:**
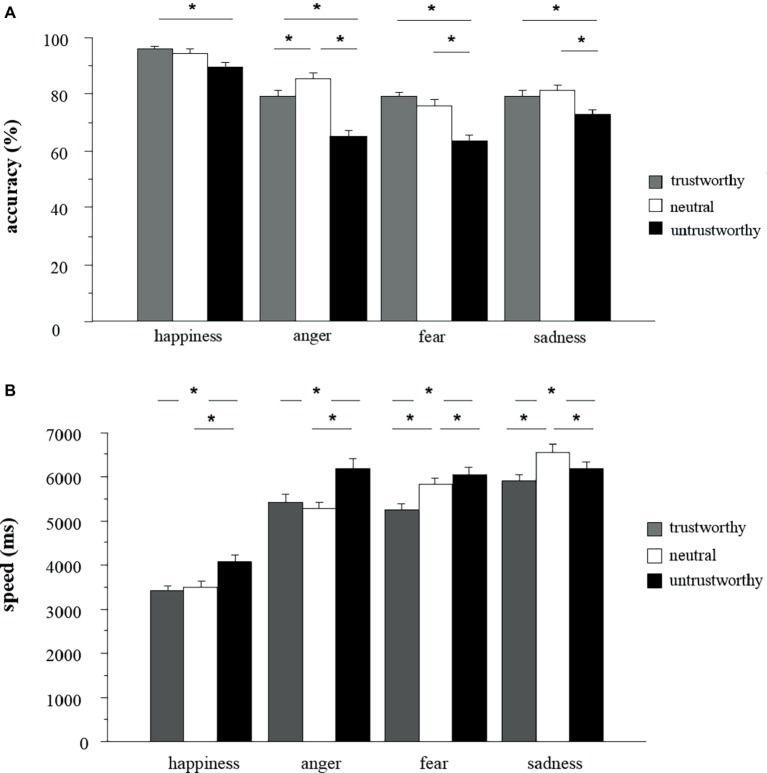
**(A)** Mean ± SE accuracy (% correct responses) in recognizing the emotions displayed by trustworthy-, neutral-, and untrustworthy-looking faces (*p < 0.05); **(B)** mean ± SE of time required to correctly recognize the emotional expressions displayed by trustworthy-, neutral-, and untrustworthy-looking faces (*p < 0.05).

Bonferroni’s test on the significant Facial Appearance × Emotion interaction, *F*(6,894) = 10.06, *p* < 0.001, $\eta_{p}^{2}=0.06,$ indicated that untrustworthy faces reduced recognition accuracy for all negative emotions compared to neutral and trustworthy faces (*p_s_* < 0.05), and they reduced recognition accuracy for happiness compared to trustworthy-looking faces (*p_s_* < 0.05). Trustworthy faces were perceived as accurately as neutral faces across emotion types (*p_s_* > 0.05), except for anger (Figure 2A).

### Speed

Consistent with the accuracy results, the type of Emotion influenced the recognition speed *F*(3,447) = 286.08, *p* < 0.001, $\eta_{p}^{2}=0.66.$ Bonferroni’s test indicated that expressions of happiness were recognized fastest (*M* = 3,761.38 ms, *SD* = 1,718.27 ms) and those of sadness (*M* = 6,327.52 ms, *SD* = 2,026.33 ms) slowest. No difference between anger (*M* = 5,770.12 ms, *SD* = 1,913.26 ms) and fear (*M* = 5,858.77 ms, *SD* = 1,913.26 ms) was found.

Recognition speed was affected by Facial Appearance *F*(2,298) = 86.47, *p* < 0.001, $\eta_{p}^{2}=0.40.$ Bonferroni’s test indicated that the emotion recognition was faster for trustworthy (*M* = 5,097.75 ms, *SD* = 2,014.37 ms) than neutral (*M* = 5,427.004, *SD* = 2,144.54; *p_s_* < 0.05) faces (*p_s_* < 0.05). In addition, untrustworthy faces (*M* = 5,763.65 ms, *SD* = 2,195.85 ms) significantly delayed emotion recognition compared to neutral and trustworthy faces.

A significant interaction Facial Appearance × Emotion, *F*(6,894) = 18.69, *p* < .001, $\eta_{p}^{2}=0.11$, was found. Bonferroni’s test indicated that untrustworthy faces slowed the recognition of happiness, anger, and sadness compared to neutral faces (*p_s_* < 0.05), leaving unaffected the recognition speed of fear (*p* > 0.05). On the other hand, trustworthy faces selectively facilitated the recognition of fear and sadness, compared to neutral faces (*p_s_* < 0.05). Trustworthy faces did not affect recognition speed of happiness (*p* > 0.05). Recognition of sadness was recognized at slowest speed when displayed by neutral faces (Figure 2B).

## Discussion

The aims of the present study were to investigate whether facial appearance-based inferences of trustworthiness affect emotion recognition and to determine whether this effect differs for specific emotions. We found that untrustworthy-looking faces induced a significant, robust disadvantage for the recognition of facial emotional cues compared to more trustworthy-looking faces. Faces that evoked negative inferences decreased accuracy recognition and delayed emotion identification, while positive inferences of trustworthiness selectively facilitated the emotion recognition speed.

Contrary to our hypothesis of an affective priming induced by trustworthiness attribution and inconsistent with studies indicating that trustworthiness inference is negatively associated with judgments of anger (Oosterhof and Todorov, 2008, 2009), we did not find a recognition advantage for happiness on trustworthy-looking faces and anger on untrustworthy-looking faces.

The overall greater effect of untrustworthy-looking faces over the trustworthy-looking ones on the recognition of emotions may be due to the tendency to perceive untrustworthy-looking faces as threatening (Oosterhof and Todorov, 2008). It is possible that faces that were perceived to have a negative valence might have captured more task-irrelevant resources than faces that were perceived to have positive valence (Schupp et al., 2004; Thomas et al., 2014), leading to a worsening of performance on the main task, namely, the recognition speed and accuracy of morphological details of emotional expressions. This interpretation might explain the lack of significant differences in emotion recognition accuracy between trustworthy and neutral faces and the reduced recognition of anger on untrustworthy-looking faces. As independent neuroimaging and behavioral neuroscience studies indicate, negatively valenced stimuli are deeply processed (LeDoux, 2000; Phillips et al., 2003), capture more attention than the neutral or friendly faces (Schupp et al., 2004; Thomas et al., 2014), and, by activating aversive/defensive responses, they may limit additional stimulus information gathering and worse behavioral performances (Pessoa, 2009).

With respect to the effects of positive trustworthiness inferences on the recognition of specific emotions, the presentation of trustworthy-looking faces facilitated the recognition of fear and sadness, emotional expressions that signal another’s distress and regulate prosocial behavior (Marsh et al., 2005, 2007; Seidel et al., 2010). However, this effect was limited to the recognition speed.

Several limitations should be noted. First, the use of a sample of healthcare professionals reduces the generalizability of the present results. An additional limitation is the use of an unequal number of positive (one: happy) and negative (three: anger, fear, and sadness) emotions, which might have contributed to a general easiness to recognize the positive emotion over the negative ones and to the lack of recognition advantage of anger on untrustworthy-looking faces. Finally, it should be noted that the facial appearance effect on the recognition of specific emotions is relatively small. Thus, additional studies are needed to determine the influence of individual (Mattarozzi, et al., 2015) and factors that may affect the recognition of specific emotions based on trustworthiness inferences.

Taken together, the present findings are in line with previous studies demonstrating that inference of trustworthiness from faces affect interpersonal interactions (Oosterhof and Todorov, 2008; Todorov et al., 2015; Mattarozzi et al., 2017) and suggest that facial appearance-based inferences, especially the negative ones, may mislead the overall ability to recognize others’ basic emotions.

Given that emotion decoding plays a key role in social interpersonal interactions and that it is at the foundation of the “soft skills” required in several work contexts (Epstein et al., 2007), such as in healthcare (Levinson et al., 2000; Delgado et al., 2017), our findings stress the importance of focusing future studies on individual and contextual determinants of emotion recognition and regulation in actual social interactions.

## Data Availability

The datasets for this manuscript are not publicly available because the data supporting the conclusions of the manuscript will be made available on request in accordance to the University of Bologna ethic rules. Requests to access the datasets should be directed to valentina.colonnello@unibo.it.

## Ethics Statement

This study was carried out in accordance with the recommendations of University of Bologna, Italy, ethical committee with written informed consent from all subjects. All subjects gave written informed consent in accordance with the Declaration of Helsinki. The protocol was approved by the University of Bologna Ethical committee.

## Author Contributions

VC conceived the study and analyzed the data. VC and KM planned and carried out the study. VC, PR, and KM contributed to the interpretation of the results and drafted the manuscript.

### Conflict of Interest Statement

The authors declare that the research was conducted in the absence of any commercial or financial relationships that could be construed as a potential conflict of interest.
